# Quantification of the Post-Fire Strength Retention Factors for Selected Standard Duplex and Lean Duplex Stainless Steel Grades

**DOI:** 10.3390/ma17020371

**Published:** 2024-01-11

**Authors:** Mariusz Maslak, Krzysztof Pancikiewicz, Michal Pazdanowski, Marek Stankiewicz, Piotr Wozniczka, Paulina Zajdel

**Affiliations:** 1 Faculty of Civil Engineering, Cracow University of Technology, Warszawska 24, 31-155 Cracow, Poland; michal.pazdanowski@pk.edu.pl (M.P.); marek.stankiewicz@pk.edu.pl (M.S.); piotr.wozniczka@pk.edu.pl (P.W.); paulina.zajdel@pk.edu.pl (P.Z.); 2 Faculty of Metals Engineering and Industrial Computer Science, The AGH University of Cracow, Pavilion A-2, Mickiewicza 30, 30-059 Cracow, Poland; krzysztof.pancikiewicz@agh.edu.pl

**Keywords:** stainless steel, standard duplex steel, lean duplex steel, post-fire strength, post-fire ductility, retention factors, recovery factors

## Abstract

The experimental quantification of retention factors related to the post-fire strength as well as the post-fire ductility of intentionally selected stainless steel grades applied in construction is the objective of the research presented here. These steel grades are characterized by a two-phase austenitic–ferritic microstructure of the duplex type. In this context, two mutually corresponding chromium–nickel–molybdenum steel grades are subjected to analysis, namely *X2CrNiMoN22-5-3* steel belonging to the standard duplex group (DSS 22% Cr) and *X2CrMnNiN21-5-1* steel belonging to the lean duplex group (LDSS). The similarities and differences in the mechanical properties exhibited by these steel grades after effective cooling, following more or less prolonged simulated fire action conforming to several development scenarios, are identified and indicated. The resistance of a given steel grade to permanent structural changes induced by the heating program proved to be the critical factor determining these properties and resulting in many cases in increased susceptibility to brittle fracture. The results obtained experimentally seem to confirm the quantitative estimates of post-fire retention factors forecast by Molkens and his team, specified for the steels exhibiting a duplex-type structure and tested by us. However, several of these estimates might be considered somewhat risky. Nevertheless, our results do not confirm the significant post-fire strengthening of steel grades belonging to the LDSS group following prior heating at a sufficiently high temperature, as reported earlier by Huang Yuner and B. Young.

## 1. Introduction—Standard Duplex versus Lean Duplex Stainless Steels Used in Construction

Duplex steels have a special place in the modern construction industry, where stainless steels are used in a wide range of applications. Their intentionally designed and properly balanced two-phase microstructure, comprising face-centered austenite and body-centered ferrite in an approximately equal ratio and crystallizing in a cubic system, determines the relatively high strength accompanied by manufacturer-warranted ductility, as expected by the user [1]. The basic difference in the chemical composition of these steels, when compared with the chemical composition typical for conventional stainless steels exhibiting an austenitic structure, is the substantially higher chromium content by weight (usually between 20 and 28%) and the increased molybdenum content (up to 5%) [2]. At the same time, the nickel content is as a rule decreased to 9%, while the nitrogen content remains at 0.05–0.50% [3]. Due to the very high corrosion resistance, in particular to the pitting corrosion initiated in chlorine-rich environments, these steels find widespread application in, among other fields, the petrochemical industry, for instance in pipeline construction or the construction of pressurized storage tanks [4].

The chemical composition of duplex steels is usually driven by the requirement to obtain thermodynamic stability of their most substantial components, i.e., austenite and ferrite, and, independently, nitrogen limit solubility. On the one hand, when the combined chromium and molybdenum content by weight in the chemical composition does not exceed 20%, one has to take into account the risk of martensitic transformation of austenite. This, as a rule, results in the hardening of the material and, as a consequence, a significant deterioration in ductility. Transitions of this type are usually related to a high susceptibility to brittle failure, occurring in an abrupt fashion, without any forewarning of gradually increasing failure risk. On the other hand, when the chromium and molybdenum content by weight in the designed steel exceeds 35%, δ-ferrite instability may occur, accompanied at a sufficiently high temperature by secondary precipitates, deleterious to its mechanical properties. A high pressure of nitrogen dissolved under these conditions in the structure of such steel may constitute an additional source of instabilities generated between individual phases [5].

As is widely known, stainless steels of the duplex type, depending on the intentionally selected chemical composition, may be divided into five basic qualitative groups. These are as follows:

1. Lean Duplex Stainless Steels—LDSS,
2. Standard Duplex Stainless Steels (with 22% Cr content)—DSS 22% Cr,
3. High-Alloyed Standard Duplex Stainless Steels (with 25% Cr content)—DSS 25% Cr,
4. Super Duplex Stainless Steels—SDSS [6,7],
5. Hyper Duplex Stainless Steels—HDSS.

The stainless steels belonging to the LDSS group, when compared with stainless steels belonging to the DSS 22% Cr group, are characterized by a significantly lower nickel and molybdenum content in their chemical composition. The reduction in the content of these elements, applied to stabilize austenite, requires the addition of manganese. This addition increases the solubility of nitrogen in the solution, and, as a consequence, increases the resistance of these steel grades to pitting corrosion [8]. However, in the case of LDSS steels, this resistance is significantly lower than the resistance exhibited by the steels belonging to the DSS 22% Cr group. The LDSS steels have been designed out of necessity as a cheaper substitute of the DSS 22% Cr steels, and have been introduced to the market driven by wide fluctuations in the price of scarcely available nickel.

The maximum corrosion resistance, as well as the best mechanical properties of duplex steels, will be achieved when the ferrite to austenite phase balance is equal to 50:50. However, in practice, achieving such a balance turns out to be difficult due to many factors, including the chemical composition of the material, the welding processes used and even the thermal history of the steel. The experimental tests carried out have shown that duplex steels classified as DSS 22% Cr have optimal corrosion resistance and mechanical properties if the ferrite content in the entire structure is maintained from 35% to 60%.

The resistance of a given steel grade exhibiting a duplex-type internal structure to pitting corrosion is usually indicated by the PREN number (pitting resistance equivalent number) determined for the considered material. This number depends directly on the chemical composition applied in practice, as it is determined by the following formula: PREN = %Cr + 3.3%Mo + 16%N. Steel grades belonging to the DSS 22% Cr group are usually characterized by a PREN number between 28 and 38, while steel grades belonging to the LDSS group are characterized by this factor remaining between 22 and 27.

## 2. Steel Grades Selected for Detailed Analysis

The research presented in this paper deals with two stainless steel grades, subjectively selected and juxtaposed for comparative purposes, exhibiting a two-phase austenitic-ferritic structure of the duplex type. The first of these grades is *X2CrNiMoN22-5-3* stainless steel belonging to the standard duplex group (DSS 22% Cr). Following its classification, this is a high-alloy, chromium–nickel–molybdenum steel. In the commercial nomenclature, this steel is denoted as 1.4462 (Werkstoffnummer). The second grade is *X2CrMnNiN21-5-1* stainless steel, also a chromium–nickel–molybdenum steel, but counted among the lean duplex steels (LDSS). In the commercial nomenclature, this steel is denoted as 1.4162 (Werkstoffnummer). The properties of both these steels are listed in detail in the code EN 10088-1 [9]. Their chemical composition is listed in Table 1.

The chemical composition of the stainless-steel samples described above was established using the Foundry Master (Worldwide Analytical Systems, Uedem, Nordrhein-Westfalen, Germany) optical emission spectrometer (OES). One may easily note that the LDSS steel selected for analysis, when compared with the DSS 22% Cr steel, is characterized by a significantly decreased nickel and molybdenum content. At the same time, it is characterized by a substantially increased manganese content.

One may easily notice that the results presented in Table 1 exhibit an overestimated carbon (C) content with respect to the values prescribed by the code. In our opinion, this is a result of using the OES spectrometer, working only within the visible spectrum, during the tests. Spectrometers of this type exhibit a tendency to adulterate the indicated content of nonmetallic elements, and in particular carbon (C) and sulphur (S). The accurate determination of the content of these elements would require an independent analysis of spectral bands within the ultraviolet range or additional verification using a LECO device.

Both stainless steel grades considered in this paper exhibit comparable mechanical properties when analyzed at room temperature. This in particular refers to the *R*_0.2_ yield limit and ultimate strength *R_m_*. These are *R*_0.2_ > 500 MPa and *R_m_* = 660–950 MPa in the case of DSS 22% Cr steel, as well as *R*_0.2_ > 480 MPa and *R_m_* = 650–850 MPa in the case of LDSS steel, respectively.

## 3. The Purpose and Scope of Conducted Research

Our research was oriented toward testing the post-fire mechanical properties exhibited by the compared steel grades. It is widely known that every steel grade subjected to the action of fire does not revert to its initial internal structure after cooling, and therefore its properties differ substantially from those exhibited prior to the fire incident. The behavior of stainless steels subjected to the simulated fire tests usually differs from the behavior exhibited by common structural steels, and in particular low-carbon steel grades [10,11]. The peculiarity of this behavior is determined by permanent changes occurring in the microstructure of the tested material when subjected to the action of high temperature and remaining after cooling [12]. However, the way these changes occur, and in particular their intensity and probability of initiation, depend to a high degree on the characteristics of the fire affecting the considered steel. The key here is not only the temperature to which the steel is heated or the heating speed [13], but also the time during which the material is subjected to the action of a constant high temperature. The situation changes diametrically, as only under these circumstances does the steel reach a temperature sufficient for the unrestrained initiation of austenitic transformation, accompanied by all more or less important repercussions resulting from the transformation of this type. The tests conducted so far indicate the strong dependence of the post-fire material properties of the considered steels on the cooling mode applied at the end of tests. When rapid cooling is applied, via the voluminous application of water spray to simulate a fire extinguishing action, the cooled material, due to local hardening, may prove to be very susceptible to brittle failure.

The first stage of testing conducted was oriented toward the identification of such fire scenarios, which could, after application to duplex steel grades selected for detailed analysis, result in unacceptable brittleness of the material [14]. For this purpose, a wide array of experimental tests were conducted, based on a series of instrumented Charpy impact tests planned and adjusted to post-fire conditions. These tests conformed to European [15,16] and US [17,18] standards. The results of these tests have been published in part and were first presented in [19,20], relating to the credibility of the testing method itself and the conclusions drawn after the application of the selected testing methodology, and later on in [21,22], taking into account various aspects of the final rating.

During the research conducted so far, the heating level of the tested samples was selected intentionally so as to enable the initiation of deleterious phase changes in the structure of the considered material, resulting in decreased ductility. The research was oriented toward discerning the sensitivity of the tested duplex steels to various types of precipitation related to these changes. The results presented in [23] were applied for this purpose. In particular, a more or less extended time of passing through the two temperature ranges was of particular interest here:

- A 475 °C brittleness zone, related to the partial change of *δ*-ferrite into spinoidal secondary *α′*-ferrite, and the precipitation of *π*, *ε* and *G* brittle phases (in the steels tested here, this phenomenon occurs in the temperature range of 300–550 °C),
- An 800 °C brittleness zone, induced by the precipitation from the solid solution (mostly *δ*-ferrite) of the carbides M_7_C_3_ and M_23_C_6_, the nitride Cr_2_N as well as the secondary phases *σ*, *χ*, *R* and *γ_2_* (in the steels tested here, this phenomenon occurs in the temperature range of 600–1050 °C).

The decreased molybdenum content by weight, characteristic for LDSS steels (with respect to an analogous content typical for steels belonging to the DSS 22% Cr group), should result in this context in [24,25,26,27,28,29,30]:

- An increased upper threshold initiation temperature limit for the 475 °C brittleness phenomenon,
- A decreased lower threshold initiation temperature limit for the 800 °C brittleness phenomenon.

The second part of these tests, completing the abovementioned tests dealing with the verification of post-fire brittleness, dealt with the determination of the post-fire mechanical properties of analyzed steels, in particular the yield limit and the tensile strength. The detailed results obtained are presented in the following section of this paper. These results seem to confirm the peculiar behavior of these steels, exhibiting a two-phase austenitic–ferritic structure of duplex type, determined in fire conditions. In this context, it qualitatively differs from that exhibited by stainless steels of a purely austenitic structure [11], as well as from that which seems to be typical of analogous steels exhibiting a purely ferritic structure [31].

The tested samples in the case of both tested steel grades were taken from hot rolled steel plates. Thus, the qualitative interpretation of numerical results obtained here should be restricted to the material subjected to this type of plastic processing. Therefore, the alternative scenario of testing post-fire mechanical properties of steels subjected to cold forming is not dealt with here. It is well known that the plastic processing method, determining the mechanical properties of steel prior to a fire episode, affects its properties determined post-fire and after cooling as well. This is discussed in [32,33], among other papers.

## 4. Sample Preparation Method and the Conducting of Tests

**Figure 1:**
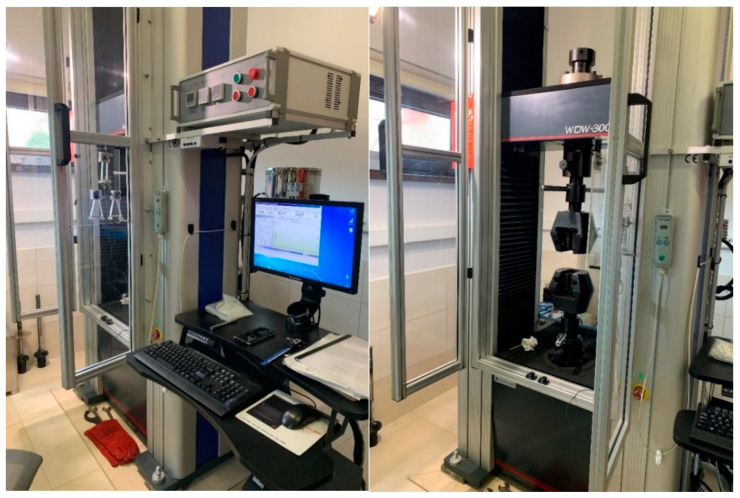
Testing setup for the static tension testing of samples cooled after surviving a simulated fire incident.

**Figure 2:**
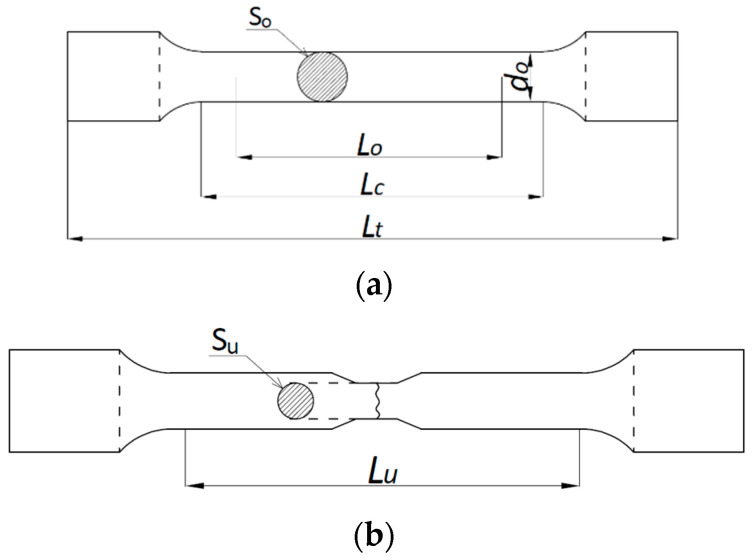
Normalized sample used for testing: (**a**) sample shape prior to rupture; (**b**) sample shape after rupture.

The experimental verification of the yield limit $f_{y,\Theta }^{post}$ and the ultimate strength limit $f_{u,\Theta }^{post}$ exhibited by duplex steels after a simulated fire incident and selected for detailed analysis was conducted on the strength testing machine WDW-300E, capable of generating a maximum tensile force of 300 kN (Figure 1). The lower index Θ denotes an earlier action of fire temperature on the tested specimen, while the upper index denotes that the indicated quantity is determined on the sample effectively cooled after surviving a simulated fire incident. The tested “fivefold” samples of normalized shape and dimensions [34] (Figure 2) were cut from hot-rolled steel plates.

**Figure 3:**
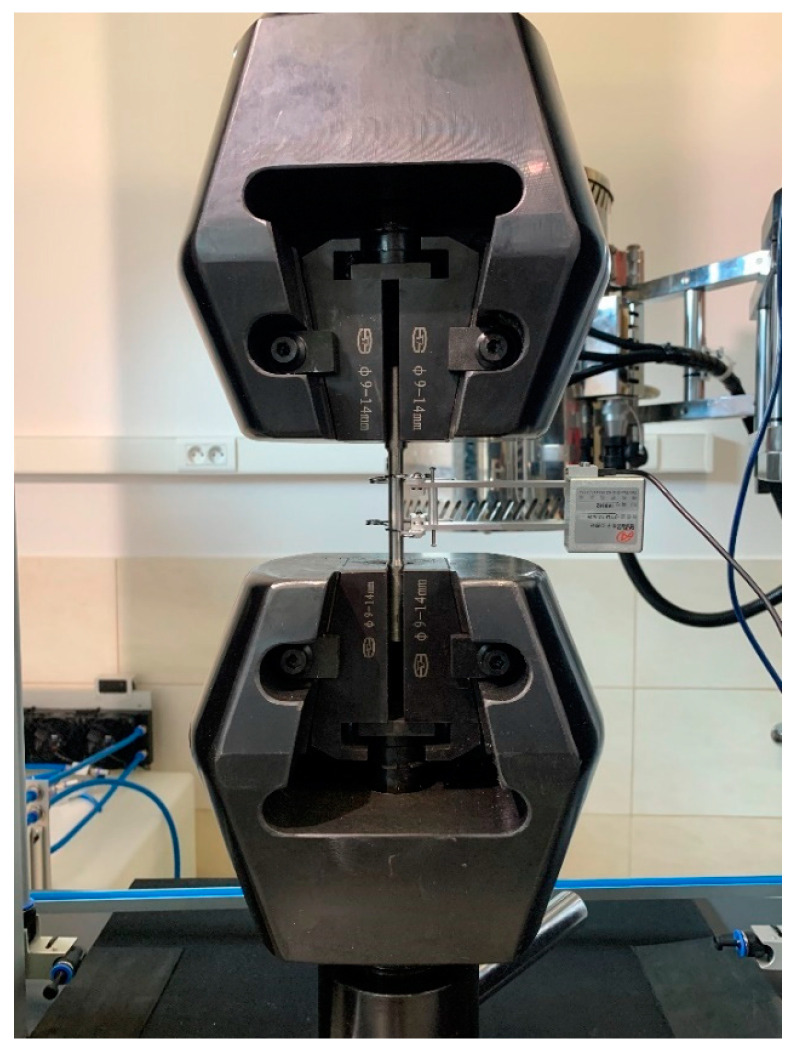
A sample in the grips of the strength testing machine. Extensometer mounting is visible.

The software included with the strength testing machine is capable of registering relations of various types, in particular the load–time, extension–time, load–extension, load–displacement or stress–relative displacement relations. Prior to each test, an initial base length *L*_0_ equal to 40 mm was marked on each sample with a scribing device with intermediate points placed every 5 mm. An extensometer with base length of 25 mm, mounted on the sample as shown in Figure 3, was used to measure the elongation. This extensometer was removed from the sample at the initiation of plastic deformation. After the conclusion of the test (i.e., after breaking the sample), the final length *L_u_* (Figure 2) was measured with an electronic caliper.

**Figure 4:**
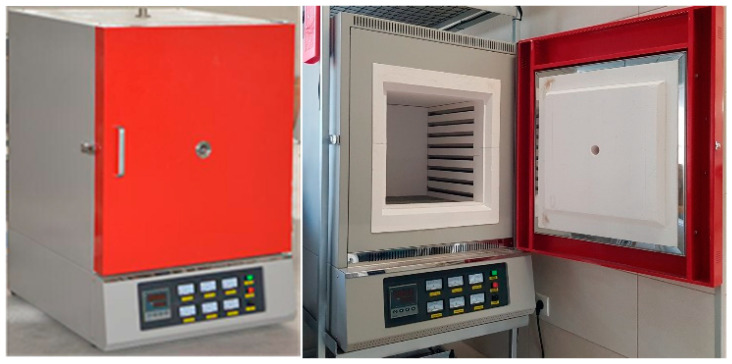
KJ-M1200-64L-IC muffle furnace used in the experiment to heat the tested samples in the simulation of a fire incident.

**Figure 5:**
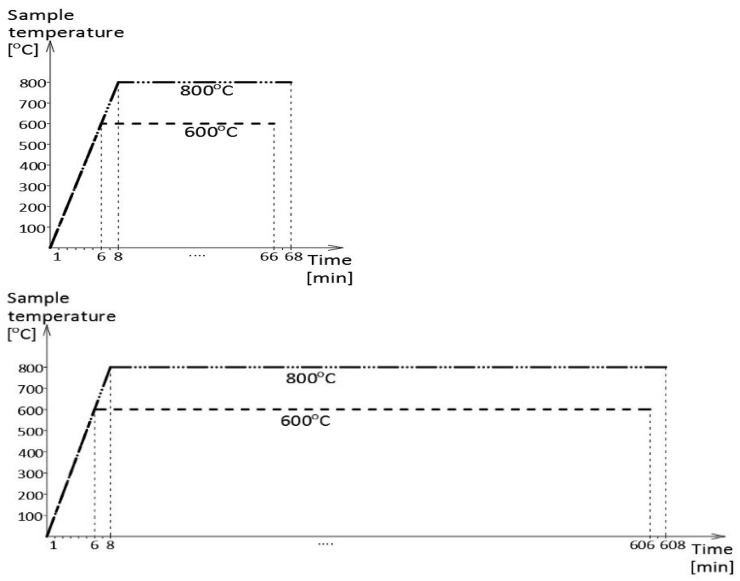
Fire simulation scenarios followed during the experiments: “short” fire on the left, “long” fire on the right.

As indicated above, the tested samples were cooled after prior action of a simulated fire incident (Figure 4). Within the scope of this simulation, several fire scenarios were considered, each modeled following the isothermal testing mode (the so-called steady-state heating regime). In particular, the following scenarios were used: a “short” fire, with the sample kept for one hour at the fire temperature, and a “long” fire, where the time spent by the sample at the fire temperature was extended to ten hours. The tested samples were heated up to the predetermined testing temperature, equal to 600 °C in the first series of tests and 800 °C in the second series, with a constant speed of 100 °C/min (Figure 5). These heating levels were selected intentionally, as the first of these levels is believed to be too low while the second is considered to be sufficiently high to induce thermally generated permanent structural changes during the tests conducted on samples made from mild carbon steel, determining their post-fire strength [12]. We decided to preserve these temperature levels during the tests conducted on samples made from stainless steels of the duplex type for comparability reasons, to preserve the ability to compare the results obtained during the research reported here with the results of other tests.

Different sample cooling modes were applied during the tests as well. In one group of tests, the samples were cooled slowly inside the laboratory furnace, to simulate the self-extinguishing of a fire, while in the other group, the samples were cooled rapidly, in water mist, to simulate the fire extinguishing action conducted by fire fighters.

Therefore, 18 samples were subjected to tensile strength testing (2 steel grades times 2 fire simulation scenarios times 2 cooling modes applied, plus for each steel grade one so-called reference sample made from the material not subjected to the action of the simulated fire temperature).

## 5. The Results Obtained and Their Interpretation

### 5.1. Sample Description Mode Applied

The results obtained during the tests are presented here on the stress–strain graphs prepared separately for each tested steel grade.

The following four-character key comprising digits and letters was used to describe the particular samples tested:

- First character (digit, 1 or 2)—denotes steel grade tested (1—*X2CrNiMoN22-5-3* steel; 2—*X2CrMnNiN21-5-1* steel);
- Second character (digit, 6 or 8)—denotes sample heating level (6—600 °C; 8—800 °C);
- Third character (letter, F or W)—denotes sample cooling mode after simulated fire incident (F—slow cooling inside the laboratory furnace; W—rapid cooling in water mist);
- Fourth character (letter, X or Y)—denotes fire simulation scenario applied (X—“long” fire duration; Y—“short” fire duration).

The *σ*–*ε* curve denoted with a single digit instead of a four letter code refers to the sample made from *X2CrNiMoN22-5-3* steel when marked with 1, while the curve denoted with 2 refers to the sample made from *X2CrMnNiN21-5-1* steel and is a typical reference curve, since it was determined while testing a sample made from an as-delivered material, i.e., not subjected to thermal action simulating a fire incident. Since this sample did not undergo heat treatment, it was not subjected to cooling.

### 5.2. Permanent Changes in the Microstructure of Tested Steels Observed in Samples Cooled after a Simulated Fire

As indicated in Section 3 of this paper, the strength tests conducted by our team were preceded by detailed analysis of the permanent changes occurring in the microstructure of the considered steel grades after the more or less prolonged action of the simulated fire varying in the scenario followed by effective cooling. A substantive discussion of the results obtained during this research has been published in [12], so here we will refer only to the most important results, as these to a large extent determine the post-fire mechanical properties of the steel grades under scrutiny, identified experimentally and presented in detail in the following section of this paper.

**Figure 6:**
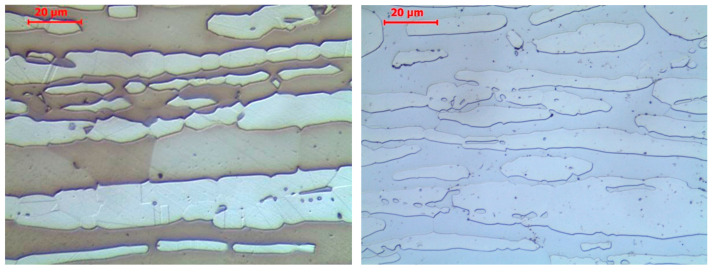
Microstructure of the analysed stainless steels observed in the as-delivered state, including: *X2CrNiMoN22-5-3* steel—**left**; *X2CrMnNiN21-5-1* steel—**right**. Light microscope pictures, magnified 200 times.

It is shown in Figure 6 that both grades of stainless steel tested in this experiment in the as-delivered state exhibited a banded two-phase structure, with alternating layers of ferrite and austenite, a phenomenon typical for duplex steels. In the case of *X2CrNiMoN22-5-3* steel, belonging to the DSS 22% Cr steels group, the ferrite content in the samples subjected to testing was determined as 65.65 ± 2.60%.

The “short fire” scenario, consisting of one-hour long heating of the samples made from *X2CrNiMoN22-5-3* steel at 600 °C, resulted in only minor permanent changes in the microstructure of the material being visible after cooling. This behaviour was evident especially when samples were cooled rapidly in water mist. The post-fire ferrite content of samples subjected to this test was determined as being 65.84 ± 3.81%. However, when the steel was left to cool inside the slowly cooling muffle furnace, infrequent precipitates of chromium nitride Cr_2_N were observed in ferrite bands. Under such circumstances, the ferrite content reduced to 61.44 ± 3.54%.

One-hour long heating of the same steel grade subject to the “short fire” scenario, but at 800 °C, resulted in structural changes of much higher intensity. Regardless of the cooling mode applied, in practice, the chromium nitride Cr_2_N precipitates were more numerous. The higher heating temperature resulted in a clearly finer structure of austenite and ferrite observed in the cooled material. In addition, under these circumstances, precipitates of different types were observed at grain boundaries as well as at the interphase boundaries between ferrite and austenite. Classical spectroscopic analysis showed the presence of precipitates rich in chromium and molybdenum. A local increase in the silicon content accompanied by a decreased nickel content was observed at these locations as well. The chemical composition determined during the tests showed that the observed precipitates probably represent a secondary phase χ with the stoichiometric formula Fe_36_Cr_12_Mo_10_ or possibly (Fe,Ni)_36_Cr_18_Mo_4_. One-hour long heating at 800 °C did not result in σ-phase precipitates, which in our opinion require the tested material to spend more time in the high-temperature environment.

The heating time was extended to ten hours under the “long fire” scenario, and when applied to the *X2CrNiMoN22-5-3* steel grade, this resulted in the further fragmentation of the material structure observed post fire. This was visible even when the heating temperature was limited to 600 °C. Regardless of the sample cooling mode applied, numerous chromium nitride precipitates were observed in this case. However, the long heating of samples at temperature this high, compared to when the temperature was restricted to 600 °C, did not result in initiating the precipitation of deleterious secondary phases.

Precipitates of this type did occur, and with significant intensity, when the “long fire” scenario was followed, with steel heated up to 800 °C. Under such a high temperature, the alloyed ferrite becomes unstable due to the high diffusion speeds of the elements dissolved in it. The high chromium and molybdenum content typical for these steels favours the development of intermetallic phases. In the ferrite band zones, besides a σ phase, the secondary austenite was created as a result of the eutectoid reaction δ→σ + γ_2_.

**Figure 7:**
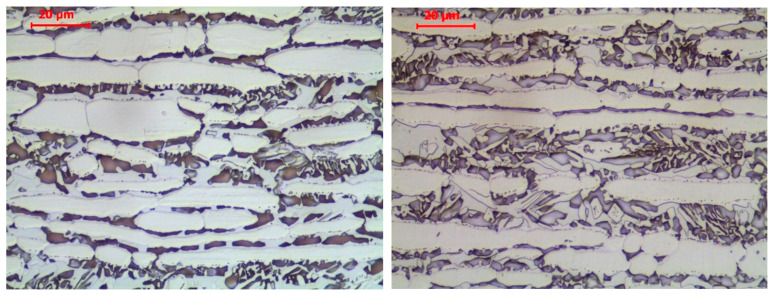
Microstructure of the *X2CrNiMoN22-5-3* steel observed in samples previously heated following the “long fire” heating regime at a temperature of 800 °C and subsequently effectively cooled. Sample cooled in muffle furnace—**left**; sample cooled in water mist—**right**. Light microscope pictures, magnified 200 times.

The post-fire microstructure of the *X2CrNiMoN22-5-3* steel grade obtained following the “long fire” scenario with a heating temperature of 800 °C is depicted in detail in Figure 7, depending on the cooling mode applied.

**Figure 8:**
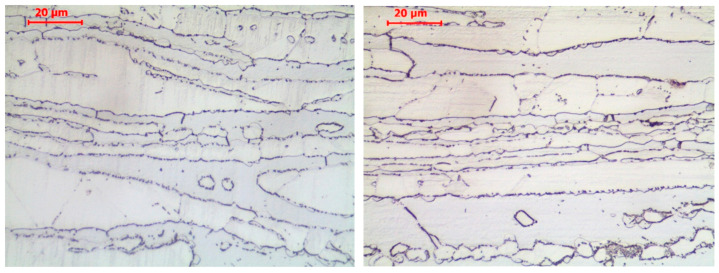
Microstructure of the *X2CrMnNiN21-5-1* steel observed on samples previously heated following the “long fire” heating regime at a temperature of 800 °C and subsequently effectively cooled. Sample cooled in muffle furnace—**left**; sample cooled in water mist—**right**. Light microscope pictures, magnified 200 times.

The observation of the permanent changes in the microstructure of the material occurring in the *X2CrMnNiN21-5-1* steel grade belonging to the LDSS steels group cooled following a simulated fire episode demonstrated the analogous character of these changes. The samples subjected to slow cooling in the muffle furnace were exposed for a longer time to a high temperature; therefore, the number of observed chromium nitride precipitates was higher. The higher heating temperature resulted in the greater fragmentation of the ferrite and austenite structure. The precipitation of deleterious secondary phases was observed at the grain boundaries and interphase boundaries of samples heated at 800 °C. However, in the case of this steel grade, the changes of this type were less intense (Figure 8). This suggests that this material is less sensitive to potential fire episodes affecting it.

### 5.3. Results Obtained for the DSS 22% Cr X2CrNiMoN22-5-3 Steel

**Figure 9:**
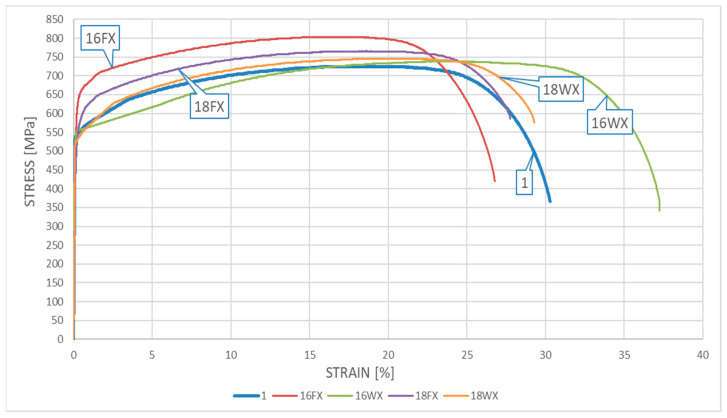
The *σ–ε* relationship obtained for samples made for *X2CrNiMoN22-5-3* steel—the “short” fire scenario.

**Figure 10:**
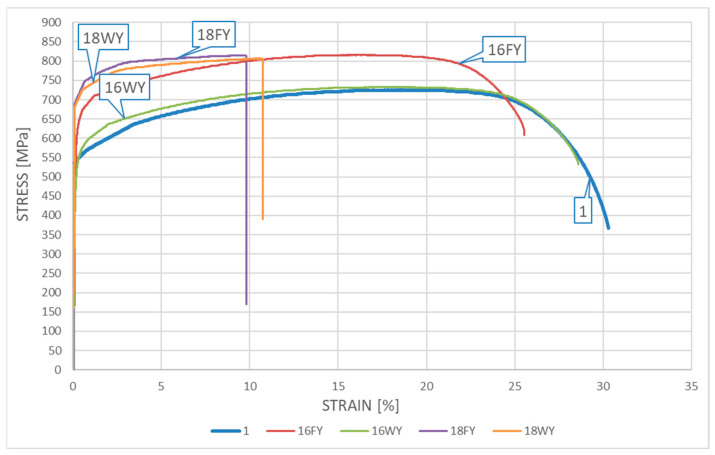
The *σ*–*ε* relationship obtained for samples made from *X2CrNiMoN22-5-3* steel—the “long” fire scenario.

The resultant *σ*–*ε* curves obtained for samples made from *X2CrNiMoN22-5-3* steel cooled down after surviving a simulated fire incident are depicted in Figure 9 for samples subjected to the simulated “short” fire and in Figure 10 for samples subjected to the simulated “long” fire.

One may easily observe on the presented graphs that after surviving a “short” fire the *X2CrNiMoN22-5-3* steel cooled slowly in the furnace exhibits significant strengthening. This phenomenon is particularly visible for the samples subjected to heating at a temperature of 600 °C (sample 16FX). It should be noted, however, that this strengthening of the material, related to partial hardening, is accompanied by a simultaneous and quite significant reduction in ductility. This ductility proved to be substantially higher when the sample heated following the same heating program was cooled down rapidly in water mist (the sample testing scenario denoted as 16WX). On the one hand, the sample heating was too low to initiate precipitation of the deleterious phases related to the 800 °C brittleness zone in the material, while on the other hand, at a sufficiently high speed of cooling, the transition time through the 475 °C brittleness zone was relatively short. Therefore, under these conditions, high ductility was not accompanied by a simultaneous strengthening of the material, as it cooled down too fast to harden. One may observe here the slight post-fire weakening of this material, related to the lower values of tensile stresses and relatively low magnitude of strains (sample 16WX).

The testing scenario related to heating the steel at 800 °C yielded qualitatively different results. Under this scenario, the material cooling method selected seemed to have a significantly smaller effect. One may compare here the graphs identified as 18FX and 18WX. The ductility of the tested steel determined after fire in general remained the same as before. The material strengthening observed post fire proved to be quantitatively insignificant as well. It seems that the qualitative differences observed here with respect to the same steel heated for the same “short” time, but at a lower temperature (only 600 °C), may be attributed to the hot material entering the area of influence of the 800 °C brittleness zone in this testing scenario. However, due to the fact that the material remained at this temperature for a relatively “short” time, this influence, albeit important, did not leave any meaningful traces of a permanent character.

The relationships obtained when testing the influence of the “long” fire scenario on this steel grade are of a similar character. Under each of the scenarios considered in detail here, the tested steel hardened substantially. The hardening was significantly more pronounced than that observed for samples subjected to the “short” fire scenario. Therefore, a long heating time proved to be the key quantitative difference, as it resulted in the heavily expressed 800 °C brittleness phenomenon. This phenomenon resulted in significant hardening of the tested steel but, at the same time, disqualified it from extended service after surviving a fire incident. This is visible on the graphs identified as 18FY and 18WY. This type of threat was not revealed during the simulation of the “short” fire scenario, as the one-hour-long heating time was too short to realize precipitation of deleterious precipitates related to the 800 °C brittleness zone in amounts capable of affecting the properties of the tested material. The higher post-fire ductility observed for the samples tested following the 16WY scenario with respect to the analogous ductility observed for samples tested following the 16FY scenario, associated with less pronounced hardening of the tested material, may be attributed to the faster transition of the cooled sample through the 475 °C brittleness zone.

A detailed juxtaposition of the experimentally determined material parameters describing the post-fire mechanical properties of *X2CrNiMoN22-5-3* steel is listed in Table 2, where $A_{t}=\frac{\Delta L_{t}}{L_{t}}\cdot 100\%$ denotes the relative elongation of the sample, measured with respect to its total length *L_t_* (Figure 2), and $A_{k}=\frac{L_{u}-L_{0}}{L_{0}}\cdot 100\%$ denotes the relative elongation of the initial measurement base, measured on the broken sample (Figure 2).

Due to the destruction mode of the samples made from this steel grade, the cross-sectional area after breaking (*S_u_*) could not be measured, and therefore it was impossible to determine the reduction in the area of cross-section *Z* (%).

### 5.4. Results Obtained for the LDSS X2CrMnNiN21-5-1 Steel

**Figure 11:**
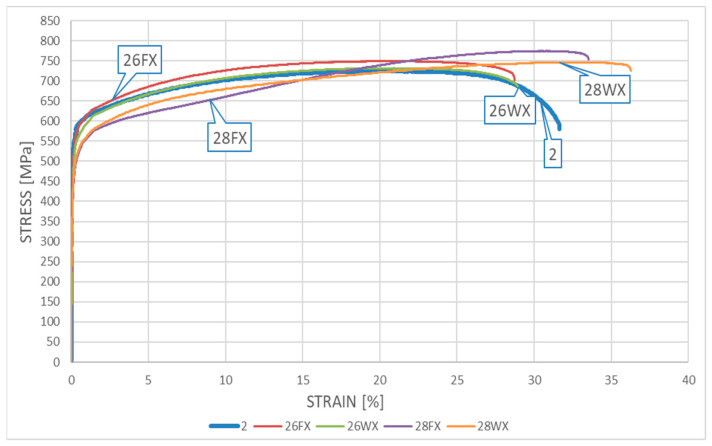
The *σ–ε* relationship obtained for samples made from *X2CrMnNiN21-5-1* steel—the “short” fire scenario.

**Figure 12:**
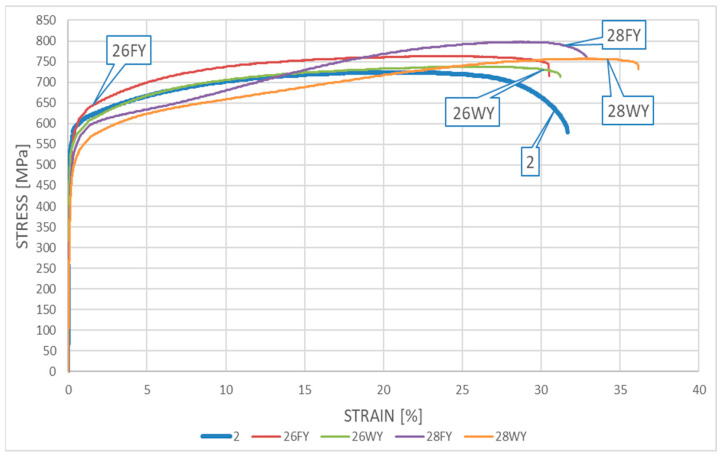
The *σ–ε* relationship obtained for samples made from *X2CrMnNiN21-5-1* steel—the “long” fire scenario.

The appropriate *σ*–*ε* relationships obtained during the tests on samples subjected to heating followed by cooling and made from *X2CrMnNiN21-5-1* steel are depicted in Figure 11 relating to the “short” fire scenario and in Figure 12 relating to the “long” fire scenario.

Detailed analysis of both of these graphs indicates the relatively small influence of the simulated fire action on the mechanical properties of the tested material determined experimentally post fire. Both the material strength and ductility in general seem to remain unaffected by the fire’s action, regardless of the fire duration applied (“short” fire versus “long” fire). The quantitative differences observed seem to be negligible from the point of view of a potential designer striving to keep the analyzed material in service after a fire incident, as these changes do not affect the capability of the material to safely resist the loads applied to it. How the sample was cooled after surviving a fire incident, the temperature at which the sample was kept, or for how long it was kept this temperature do not seem to be important. One may even dare to say that under several of the testing scenarios applied, the post-fire mechanical properties of the tested material slightly improved when compared with the same properties determined for the samples in the “as-manufactured” condition, i.e., not affected by the simulated fire action.

In general, the weak influence of fire incident duration and intensity on the registered post-fire strength and ductility clearly distinguishes the LDSS steel from the DSS 22% Cr steel described above. This is a consequence of a shift to the right on the TTT (Time–Temperature–Transformation) graph of the temperature range related to the 800 °C brittleness. Thus, from a practical point of view, the application of LDSS steel seems to be safer when compared with SDSS steel, if only the risk of prior exposure to fire is considered.

A detailed juxtaposition of the experimentally determined material parameters describing the post-fire mechanical properties of *X2CrMnNiN21-5-1* steel is listed in Table 3. The sample destruction mode, analogous to that observed for samples made from *X2CrNiMoN22-5-3* steel, precluded the measurement of the cross-sectional area after breaking. As a result, the value of parameter *Z* (%), identifying the degree of sample necking, could not be determined.

## 6. Quantification of Post-Fire Recovery Coefficients Observed on the Strength and Ductility of Tested Steels

**Figure 13:**
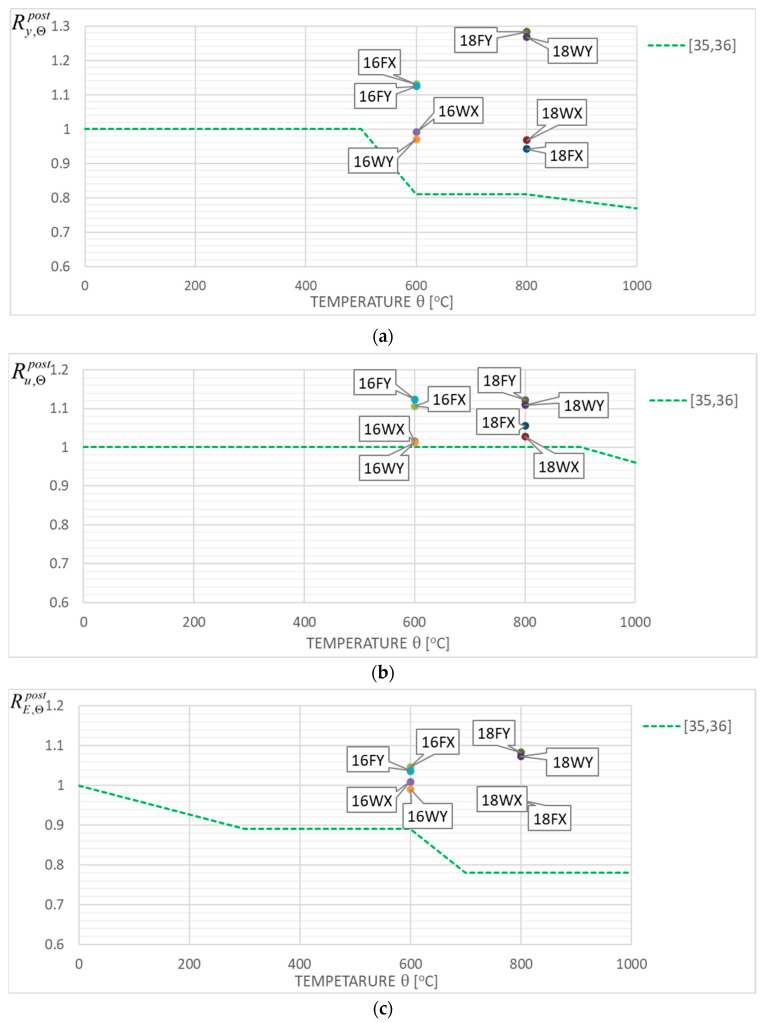

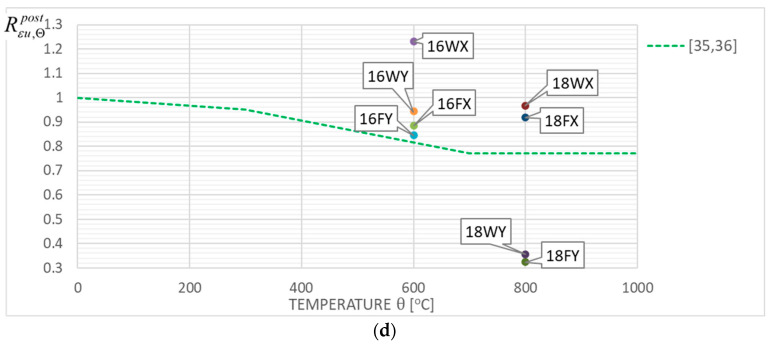
Post-fire strength and ductility retention factors obtained during tests on the *X2CrNiMoN22-5-3* stainless steel grade juxtaposed with the values recommended in [35,36], (**a**)—yield limit, (**b**)—ultimate strength, (**c**)—linear elasticity modulus, (**d**)—total strain.

**Figure 14:**
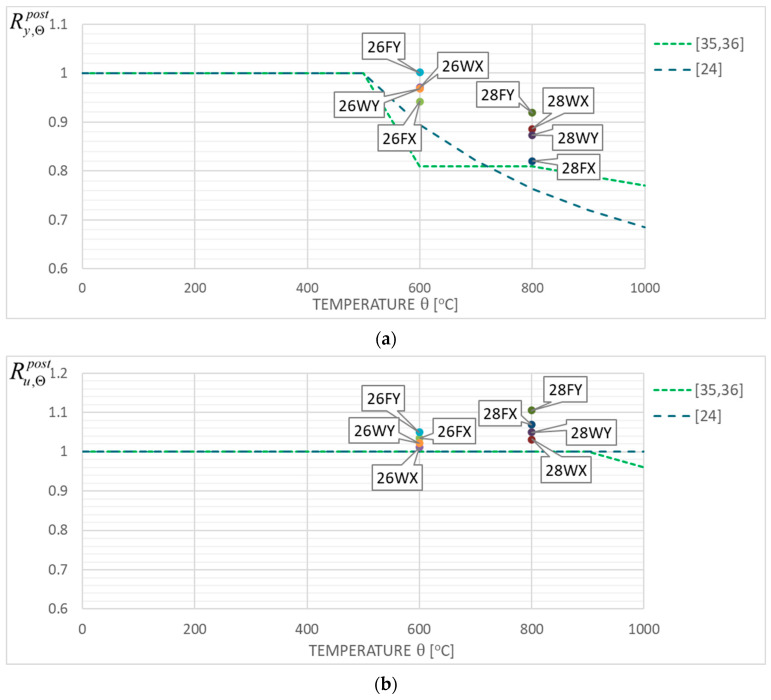

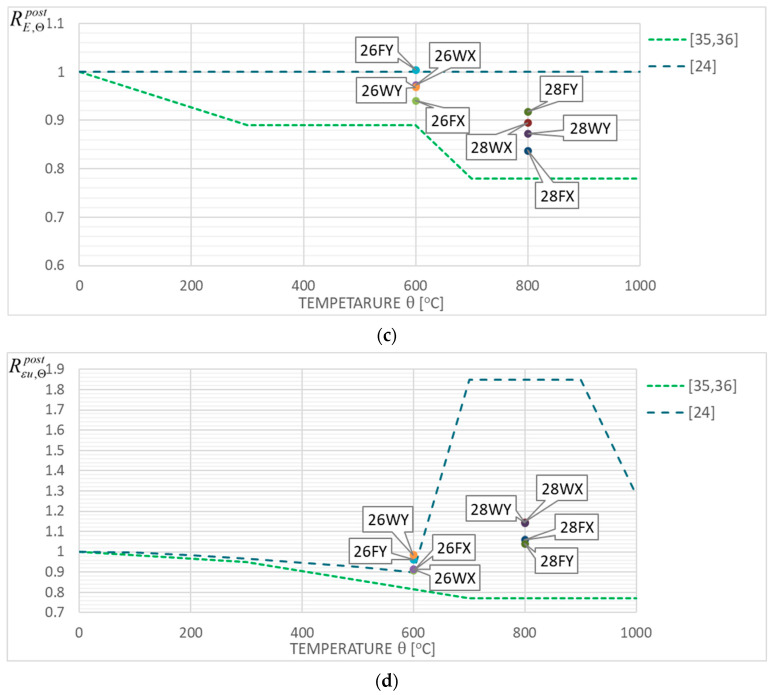
Post-fire strength and ductility retention factors obtained during tests on the *X2CrMnNiN21-5-1* stainless steel grade juxtaposed with the values recommended in [24] as well as in [35,36], (**a**)—yield limit, (**b**)—ultimate strength, (**c**)—linear elasticity modulus, (**d**)—total strain.

The coefficients quantifying permanent changes in a given quantity determined for the cooled material after a fire episode when compared with its initial value characterizing the considered material before simulated fire exposure constitute an a posteriori recovery measure of the strength and ductility observed for the stainless steels tested here. Therefore, the following quotients, identified in the professional bibliography as retention factors referring to the yield limit ($R_{y,\Theta }^{post}=\left(f_{y,\Theta }^{post}/f_{y,20}\right)$), the ultimate tensile strength ($R_{u,\Theta }^{post}=\left(f_{u,\Theta }^{post}/f_{u,20}\right)$), the linear modulus of elasticity ($R_{E,\Theta }^{post}=\left(E_{a,\Theta }^{post}/E_{a,20}\right)$) and the limit strain of tested steel sample resulting in fracture ($R_{\varepsilon u,\Theta }^{post}=\left(\varepsilon_{u,\Theta }^{post}/\varepsilon_{u,20}\right)$), were used here. The detailed values of these factors obtained during the tests reported here are listed in Table 4 and Table 5, separately for each steel grade tested. These results are also shown in Figure 13a–d for the *X2CrNiMoN22-5-3* steel and in Figure 14a–d for the *X2CrMnNiN21-5-1* steel, with each test result attributed to the testing scenario indicated by a symbol explained in detail in the Section 5.1 of this paper. The graphs mentioned here the depict retention factors obtained experimentally in our research, related to their values recommended for practical application, juxtaposed in [35,36] for steels of a duplex two-phase internal structure as a result of research by Molkens and his team. For *X2CrMnNiN21-5-1* steel, belonging to the LDSS group, these data, presented in Figure 14a–d, were completed according to the alternative recommendations originating in [24], as these recommendations were calibrated by taking into account the specificity of this material. It should be underlined, however, that all of these values mentioned above and recommended for practical application are interpreted as appropriate quantiles of the material properties interpreted as random variables. Thus, the probability of their actual underestimation, accepted by the user of a given building that is to be used after a fire, is set at an intentionally low level.

The yield limit of steel *f_y_* is understood on these graphs as a conventional value, listed at the *R*_0.2_ stress level. The value of limit strain *ε_u_* was determined based on the measured elongation *A_t_* listed in detail in Table 2 and Table 3, to preserve the compliance with the scale used on the horizontal axis in Figure 9, Figure 10, Figure 11 and Figure 12.

The results of the research reported here seem, to a large extent, to positively conform to the recommendations contained in [35,36], as they are located in general on the safe side when compared with the bottom limit values contained therein. This remark pertains not only to the post-fire strength of tested steel, but also to its post-fire ductility. The significantly reduced capacity for plastic strain exhibited by a sample previously heated for a sufficiently long time at a temperature of 800 °C, regardless of the cooling mode applied (as shown for the samples denoted as 18FY and 18WY in Figure 13d, and, independently, in Figure 7), seems to be a notable exception here. Such behavior, due to the inherent risk of brittle failure, unequivocally excludes the capacity for the extended use of this steel grade and in particular for safe load-bearing service after a fire. It is clearly visible that in many fire development scenarios which may occur in real life, the tested steel does not fully recover its strength after surviving a fire incident followed by effective cooling. However, in many other scenarios, the mechanical properties of this steel visibly improve, and this may justify the absence of recommendations to apply an appropriate reduction in limit tensile strength value and an assumption of $R_{u,\Theta }^{post}=1.0$, as depicted in Figure 13b and Figure 14b. In this context, the recommendation contained in [35,36] pertaining to the reduction in the value of the linear elasticity coefficient (Figure 13c and Figure 14c) seems to be very safe as well.

However, our research reported here did not confirm such a clear improvement in the post-fire ductility exhibited by the *X2CrMnNiN21-5-1* steel belonging to the LDSS group and effectively cooled after simulated fire exposure at 800 °C, as the authors of [24] (Figure 14c) would like to see.

An alternative approach to describe the dependence of the post-fire steel mechanical properties’ reduction factor on the maximum temperature at which the steel was heated was proposed in [36]. The reduction in the *i*-th property in this approach is still measured by the retention coefficient $R_{i,\Theta }^{post}$, but this time it is interpreted as a product of the code reduction coefficient $k_{y,\Theta }$, $k_{u,\Theta }$ and $k_{E,\Theta }$, respectively, determined as for the fire conditions based on Appendix C of the code [37], and a corresponding recovery factor $r_{y,\Theta }^{post}$, $r_{u,\Theta }^{post}$ and $r_{E,\Theta }^{post}$, specified for post-fire conditions. The recovery factors $r_{i,\Theta }^{post}$ in this method are determined as a quotient of the *i*-th value determined on a sample cooled after a fire and an appropriately reduced value of the same property determined at a given heating time under the assumption of fire scenario. Therefore, for example, $R_{y,\Theta }^{post}=\frac{f_{y,\Theta }^{post}}{f_{y,20}}=k_{y,\Theta }\cdot r_{y,\Theta }^{post}=\frac{f_{y,\Theta }^{fire}}{f_{y,20}}\frac{f_{y,\Theta }^{post}}{f_{y,\Theta }^{fire}}$. A juxtaposition of the recovery factors obtained during the research presented here accompanied by the information on the sample heating and cooling scenarios applied during the tests is presented in Table 6 and Table 7.

## 7. Concluding Remarks

Our research has shown quite significant differences in the post-fire mechanical properties exhibited by the compared stainless steel grades. In spite of the fact that *X2CrNiMoN22-5-3* steel belonging to the LDSS class due to its intentionally selected chemical composition is usually treated as a cheaper substitute for *X2CrNiMoN22-5-3* steel belonging to the DSS 22% Cr class, it proved to be more resistant in the context of the post-fire brittleness phenomenon. This type of risk is particularly important for stainless steels exhibiting a two-phase austenitic–ferritic microstructure of the duplex type. This structure is usually associated with the potential capacity to reveal harmful precipitates in the structure of the material, negatively affecting its strength and ductility. This precipitation process usually intensifies in two temperature ranges—at a high temperature, the so-called 800 °C brittleness, and at a lower temperature, the so-called 475 °C brittleness. The extent to which passing through both these ranges permanently weakens the given steel grade during the cooling process and these changes persist after the steel attains an ambient temperature depends mostly on the time spent passing through both of these ranges. Therefore, the rapid cooling of the tested samples in water mist, significantly speeding up the cooling process, usually proves to be more advantageous when compared with traditional slow cooling in a muffle furnace, modeling self-extinguishing of the fire. However, overly rapid cooling may result in the local hardening of the material. This will result in strengthening, but at the expense of a substantial increase in the susceptibility to brittle failure.

It has to be underlined that during the research reported here, the 800 °C brittleness zone was reached only when following the scenarios where the tested samples were heated to the temperature enabling austenitic transformation. When following the “short” fire scenario, the samples were kept at this temperature for a time which was too short to fully reveal the deleterious phenomena related to the 800 °C brittleness. Only after this time had been extended to 10 h, as in the “long” fire scenario, was it possible to observe the full-scale results of the coincidence of both of the phenomena described above.

Of all of the testing scenarios applied during our research, only those denoted as 18FY and 18WY resulted in a material state after the simulated fire excluding its capacity to safely resist the loads applied. Let us note, however, that under those scenarios, the cooling mode applied proved to be unimportant. The full-scale brittleness revealed itself in these cases as a direct result of heating to a sufficiently high temperature, initiating austenitic transformation in its internal structure, followed by keeping the hot material at this temperature for a time which was long enough to fully reveal the deleterious results of the 800 °C brittleness phenomenon.

Both the retention factor values $R_{i,\Theta }^{post}$, listed in this paper in Table 4 and Table 5, as well as the correlated values of the recovery factors $r_{i,\Theta }^{post}$, listed here in Table 6 and Table 7, provide the potential designer, deciding whether to further use a given steel grade to safely support the loads applied to it after a fire, with the opportunity to assess to what extent this steel grade retained its initial strength and ductility under these circumstances. Of course, the values assigned to the recovery factors in this approach are substantially higher than those assigned to the retention factors, as the former refer to material properties significantly deteriorated at the fire temperature, instead of those characterizing it prior to the fire incident and thus not subject to reduction.

Detailed analysis of these factors determined during our experiments leads to the conclusion that for both steel grades tested, the ultimate strength $f_{u,\Theta }^{post}$ determined after the simulated fire in each of tested simulated fire scenario episodes was not only fully preserved, but even slightly increased. This conclusion does not hold in the case of the conventional yield limit $f_{y,\Theta }^{post}$, but the relative reduction in this value, as determined during the experiment, does not seem to be computationally relevant. Let us note, however, that when this evaluation criterion is applied, the degree to which it is permanently reduced, determined for the material cooled after surviving an a priori fire incident, seems to be more pronounced when a steel grade belonging to the LDSS class is considered.

In general, the $R_{i,\Theta }^{post}$ retention factor values close to 1.0 obtained during our experiments for both steel grades indicate that the possible further use of these steel grades after fire is restricted by a qualitative understanding of the possible risk of full-scale brittleness effects being revealed in the material, as the risk of brittle failure seems to constitute the critical factor here. As shown above, such a critical scenario has been identified and discussed. The value of $R_{\varepsilon u,\Theta }^{post}$ related to this scenario indicates that only 32–36% of the initial material ductility has been recovered (Table 4). Interestingly, as mentioned earlier, this type of threat was not identified for steel classified as LDSS (Table 5) in the fire scenarios analyzed.

Figure 13 and Figure 14 included in this paper confirm that the recommendations presented in [35,36] and interpreted as appropriate quantiles of particular values treated as random variables have been calibrated generally in a safe way, though, in certain cases, in a somewhat risky manner. The proposals for such calibrations found in [24] seem not to be confirmed in the view of our research. This is particularly visible in Figure 14d.

## Figures and Tables

**Table 1:** Chemical composition of stainless steel grades selected for detailed analysis (% by weight).

Element	*X2CrNiMoN22-5-3* Steel—*SDSS* Type	*X2CrNiMoN22-5-3* Steel—Admissible Values (according to [9])	*X2CrMnNiN21-5-1* Steel— *LDSS* Type	*X2CrMnNiN21-5-1* Steel— Admissible Values (according to [9])
C	0.0507	<0.03	0.0707	<0.03
Si	0.266	<1.0	0.670	<1.0
Mn	1.8	<2.0	4.25	4.0–6.0
P	0.027	<0.035	0.0315	<0.040
S	<0.005	<0.015	0.0111	<0.03
Cr	23.7	21–23	20.6	21.0–22.0
Ni	4.74	4.5–6.5	1.70	1.35–1.70
Mo	2.92	2.5–3.5	0.303	0.10–0.80
Ti	0.0082	-	0.0115	-
Cu	0.184	-	0.291	0.10–0.80
Al	0.0097	-	0.0125	-
Co	0.0622	-	0.291	-
Nb	0.0056	-	0.0061	-
V	0.0385	-	0.0626	-
W	<0.02	-	<0.02	-
N	-	0.10–0.22	-	0.20–0.25

**Table 2:** Post-fire mechanical properties of the *X2CrNiMoN22-5-3* steel determined during the static tensile test.

Sample Identification	R_0.2_ (MPa)	R_m_ (MPa)	A_t_ (%)	A_k_ (%)
1	537	726	30.3	34.1
16FX	607	803	26.8	32.8
16WX	533	737	37.3	37.8
18FX	506	766	27.8	30.6
18WX	520	746	29.3	32.8
16FY	604	816	25.6	29.3
16WY	521	735	28.6	34.3
18FY	689	815	9.8	5.3
18WY	681	805	10.8	5.6

**Table 3:** Post-fire mechanical properties of the *X2CrMnNiN21-5-1* steel determined during the static tensile test.

Sample Identification	R_0.2_ (MPa)	R_m_ (MPa)	A_t_ (%)	A_k_ (%)
2	528	724	31.7	37.0
26FX	497	749	28.8	33.5
26WX	513	732	29.0	33.4
28FX	433	774	33.6	33.9
28WX	468	746	36.3	38.3
26FY	529	760	30.5	33.4
26WY	511	740	31.2	33.8
28FY	485	800	33.0	34.5
28WY	461	760	36.2	38.8

**Table 4:** Values of particular retention factors obtained experimentally for *X2CrNiMoN22-5-3* steel belonging to the DSS 22% Cr class, based on the fire exposure scenario applied.

Fire Exposure Scenario Followed during the Experiment	$R_{y,\Theta }^{post}$	$R_{y,\Theta }^{post}$	$R_{E,\Theta }^{post}$	$R_{\varepsilon u,\Theta }^{post}$
16FX	1.13	1.11	1.05	0.88
16WX	0.99	1.02	1.01	1.23
18FX	0.94	1.06	0.96	0.92
18WX	0.97	1.03	0.99	0.97
16FY	1.12	1.12	1.04	0.84
16WY	0.97	1.01	0.99	0.94
18FY	1.28	1.12	1.08	0.32
18WY	1.27	1.11	1.07	0.36

**Table 5:** Values of particular retention factors obtained experimentally for *X2CrMnNiN21-5-1* steel belonging to the LDSS class, based on fire exposure scenario applied.

Fire Exposure Scenario Followed during the Experiment	$R_{y,\Theta }^{post}$	$R_{y,\Theta }^{post}$	$R_{E,\Theta }^{post}$	$R_{\varepsilon u,\Theta }^{post}$
26FX	0.94	1.03	0.94	0.91
26WX	0.97	1.01	0.97	0.91
28FX	0.82	1.07	0.84	1.06
28WX	0.89	1.03	0.90	1.15
26FY	1.00	1.05	1.00	0.96
26WY	0.97	1.02	0.97	0.98
28FY	0.92	1.10	0.92	1.04
28WY	0.87	1.05	0.87	1.14

**Table 6:** Values of particular recovery factors obtained experimentally for *X2CrNiMoN22-5-3* steel, based on the fire exposure scenario applied.

Fire Exposure Scenario Applied during the Experiment	$r_{y,\Theta }^{post}$	$r_{u,\Theta }^{post}$	$r_{E,\Theta }^{post}$
16FX	2.69	1.98	1.38
16WX	2.36	1.81	1.33
18FX	6.28	4.80	1.53
18WX	6.46	4.67	1.57
16FY	2.68	2.01	1.36
16WY	2.31	1.81	1.30
18FY	8.55	5.10	1.72
18WY	8.45	5.04	1.70

**Table 7:** Values of particular recovery factors obtained experimentally for *X2CrMnNiN21-5-1* steel, based on the fire exposure scenario followed.

Fire Exposure Scenario Applied during the Experiment	$r_{y,\Theta }^{post}$	$r_{u,\Theta }^{post}$	$r_{E,\Theta }^{post}$
26FX	2.24	1.85	1.24
26WX	2.31	1.81	1.28
28FX	5.47	4.86	1.33
28WX	5.91	4.68	1.42
26FY	2.39	1.87	1.32
26WY	2.30	1.83	1.27
28FY	6.12	5.02	1.46
28WY	5.82	4.77	1.39

## Data Availability

Data are contained within the article.
